# Successful Re-administration and Extended Dosing of Brentuximab Vedotin Monotherapy for Relapsed Anaplastic Large Cell Lymphoma in a Vulnerable Patient

**DOI:** 10.7759/cureus.84058

**Published:** 2025-05-13

**Authors:** Tomoki Minemura, Shohei Kikuchi, Takuma Fujihira, Yoshimi Nabe, Ryusuke Horaguchi, Yusuke Kamihara, Akinori Wada, Kento Ono, Tsutomu Katou, Tsutomu Sato

**Affiliations:** 1 Department of Hematology, Toyama University Hospital, Toyama, JPN; 2 Department of Internal Medicine, Toyama Nishi General Hospital, Toyama, JPN

**Keywords:** brentuximab vedotin-induced neuropathy, brentuximab vedotin treatment, cd30+ anaplastic large cell lymphoma, chemotherapy-induced peripheral neuropathy, geriatric assessment, vulnerable patients

## Abstract

Brentuximab vedotin (BV) monotherapy has significant clinical efficacy against relapsed anaplastic large cell lymphoma. While BV monotherapy re-administration and extended duration of treatment show therapeutic potential, particularly in patients ineligible for autologous hematopoietic stem cell transplantation or conventional salvage chemotherapy due to advanced age or poor performance status, long-term administration is frequently limited by cumulative neurotoxicity. Herein, we present a case of successful BV monotherapy re-administration and extended treatment over 4.3 years in a vulnerable patient. This therapeutic approach expands treatment options available for elderly patients and those with poor performance status.

## Introduction

The achievement of sustained complete remission (CR) by induction chemotherapy in peripheral T-cell lymphoma remains limited, particularly in elderly patients aged >60 years or those with poor ECOG performance status (PS) (≥2) [1]. For these patients, although effective secondary treatment is essential, clinical outcomes have been poor for autologous hematopoietic cell transplantation (auto-HCT) ineligible relapsed cases, with positive responses to conventional salvage chemotherapy seen only in patients maintaining good PS (0-1) at relapse [1,2]. Brentuximab vedotin (BV), an anti-CD30 antibody-conjugated monomethyl auristatin E (MMAE), monotherapy has demonstrated high clinical efficacy as a salvage therapy for relapsed anaplastic large cell lymphoma (ALCL) [3,4]. It is noteworthy that some patients who achieved CR maintained long-term remission with BV monotherapy alone, suggesting the possibility of sustained remission without auto-HCT [4].

The introduction of BV monotherapy has significantly improved this situation by expanding treatment options for transplant-ineligible patients. However, the optimal treatment duration remains to be determined. In addition, long-term BV administration is frequently limited by neurological complications. A key trial reported that peripheral neuropathy occurred in more than half of the patients, typically manifesting early during treatment [4]. Given BV monotherapy's remarkable efficacy and its therapeutic potential for re-administration and long-term use in patients with transplant-ineligible and poor PS, the management of peripheral neuropathy presents a significant clinical challenge.

In this report, we present a successful case of re-administration and long-term dosing of BV monotherapy for relapsed ALCL with poor PS, where long-term treatment for approximately 4.3 years was achieved.

## Case presentation

A 70-year-old female presented with advanced-stage systemic ALK-negative ALCL, which progressed from a primary cutaneous ALCL diagnosed in her 40s. The patient had previously attained a complete response after eight cycles of pirarubicin, cyclophosphamide, doxorubicin, vincristine, and prednisolone (THP-COP) therapy but subsequently noticed enlarged lymph nodes in the left groin two years after the initial treatment.

**Figure 1:**
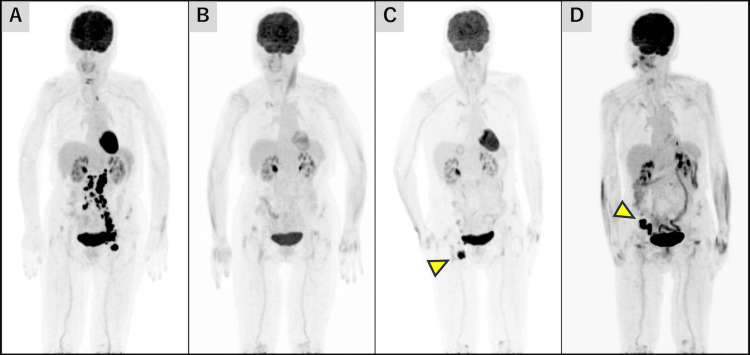
PET imaging findings after the initial relapse Maximum intensity projection (MIP) imaging of 18^F^ FDG-PET demonstrates sequential disease status prior to re-administration of brentuximab vedotin: at initial relapse (A), following completion of eight cycles of brentuximab vedotin monotherapy (B), at secondary relapse (C), and post-radiation therapy (D). Yellow arrows indicate sites of the abnormal accumulation of lymph node lesions.

Computed tomography (CT) and 18-fluorodeoxyglucose positron emission tomography (18^F^ FDG-PET) scans revealed lesions in the left groin, both common iliac and para-aortic lymph nodes, confirming disease recurrence (Figure 1A).

Subsequently, BV monotherapy at a full dose of 1.8 mg/kg was administered as second-line therapy. The patient exhibited a rapid clinical response, and left inguinal lymphadenopathy showed a significant reduction. After eight cycles of BV monotherapy, the patient achieved a complete metabolic response, as assessed by 18^F^ FDG-PET (Figure 1B). Due to comorbidity disease diffuse idiopathic skeletal hyperostosis (DISH), the patient required wheelchair assistance and exhibited PS 3, and she had been maintained on long-term duloxetine therapy for chronic lower back pain. The other patient's comorbidities included essential tremor and anxiety neurosis. The patient had been receiving long-term treatment with the beta-blocker propranolol and the benzodiazepine medications alprazolam and brotizolam. To mitigate potential adverse effects on activities of daily living (ADL) caused by neurotoxicity, BV therapy was discontinued after eight cycles, and the patient was placed under observation.

One year after BV monotherapy initiation, the patient underwent surgical treatment for a lumbar compression fracture. During postoperative rehabilitation, right inguinal lymph node enlargement was detected. 18^F^ FDG-PET-CT demonstrated isolated uptake in the right inguinal lymph nodes (Figure 1C). Based on the patient's ADL and the presence of a localized lesion, radiation therapy (40 Gy/20 sessions) was administered. Post-radiation 18^F^ FDG-PET-CT revealed new lymph node enlargement around the right common iliac vessels in the non-irradiated field, suggesting treatment failure (Figure 1D).

Considering systemic lesions, systemic chemotherapy was considered appropriate. Given concerns regarding potential deterioration in PS after spinal surgery, a comprehensive geriatric assessment (CGA) was performed. The assessment revealed the following: Geriatric-8 (G8) score of 10 points (below the threshold of 14 points), indicating positive screening and nutritional deficiency; Charlson Comorbidity Index (CCI) of 0 points, suggesting no significant comorbidities; Lawton-instrumental activities of daily living scale (IADL) score of 3/8, indicating impairment in shopping, food preparation, housekeeping, laundry, and transportation; and Mini-Cog examination score of 5/5, demonstrating intact cognitive function [5,6]. Despite the low PS, the patient maintained preserved organ function and cognitive status. Considering previous favorable responses to BV, re-administration was considered the optimal therapeutic option. The treatment protocol specified continuation of therapy until either disease progression or treatment-limiting adverse events were observed.

**Figure 2:**
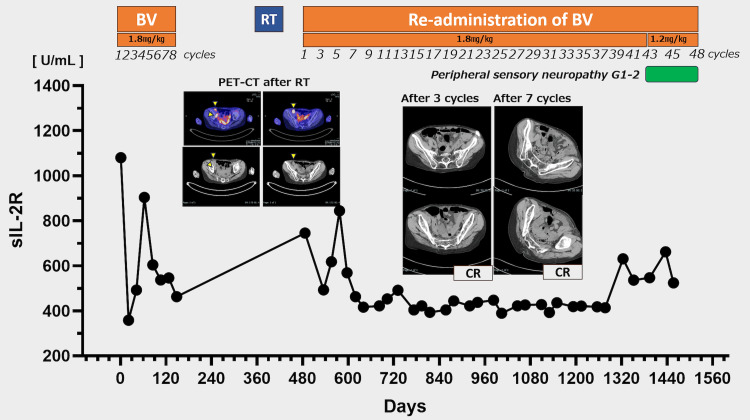
Clinical course of brentuximab vedotin administration. During both the initial administration and re-administration of BV, serial measurements of soluble interleukin-2 receptor (sIL-2R) demonstrated a progressive decline. Following re-administration, CT evaluation revealed CR after three cycles. Regular surveillance CT examinations performed at three-month intervals confirmed sustained remission status throughout the extended treatment period.

Upon re-administration of BV monotherapy at a dose of 1.8 mg/kg, CR was documented through CT assessment after three additional cycles (Figure 2). The patient maintained CR with no significant adverse events during regular quarterly CT evaluations for approximately three years. Following cycle 42 (total of 50 cycles), the patient developed grade 1-2 peripheral neuropathy, requiring a dose reduction to 1.2 mg/kg from cycle 43. In addition, mirogabalin was initiated at 10 mg per day; however, dose escalation was not feasible due to severe somnolence, and the patient showed no improvement in peripheral neuropathy. Despite protocol modification to a four-week dosing interval from cycle 45, grade 2 peripheral neuropathy persisted. During long-term brentuximab vedotin administration, the patient maintained a good quality of life and remained independent while receiving nursing care services. However, cognitive decline manifested, particularly memory impairment, which preceded the onset of peripheral neuropathy. Treatment was discontinued following patient transfer to an alternative care facility, and therapy was concluded after 48 cycles, with transition to the best supportive care. In total, brentuximab vedotin was administered for 48 cycles over three years after re-administration, resulting in 56 cycles over approximately 4.3 years from the initial administration.

## Discussion

BV monotherapy has demonstrated remarkable clinical efficacy as a salvage therapy for relapsed ALCL, with 86% (50 of 58 cases) of patients showing an objective response rate (ORR) and 57% (33 of 58 cases) achieving CR [3]. Among patients who achieved CR, BV demonstrated 79% overall survival (OS) and 57% progression-free survival (PFS) at five years, with median response duration not reached [4]. Of particular significance, some patients achieving CR maintained long-term remission with BV monotherapy alone, indicating the possibility of long-term remission without auto-HCT [4]. Re-administration of BV in ALCL exhibited favorable secondary responses with manageable toxicity profiles comparable to those after initial administration [7,8]. Specifically, Bartlett et al. documented an ORR of 88% and a CR of 63% in eight patients with relapsed ALCL, whereas Fukuhara et al. reported an ORR of 70% with a CR of 60% in 10 patients. Both studies revealed sustained remission among complete responders, with a median PFS of 12.9 months in Bartlett's study and not reached at analysis in Fukuhara's study [7,8]. Foyil et al. described a case of multiple BV treatments in a 36-year-old male patient with relapsed ALCL after auto-HCT [9]. The patient achieved second to fourth CR through three separate courses of BV monotherapy, receiving 40 total cycles over three years [9]. Although patients with PS ≥2 were not evaluated in the clinical trial, the clinical efficacy of initial and re-treatment studies and the advantages of mono-treatment in terms of tolerability indicate that BV monotherapy may be a preferred therapeutic option for auto-HCT ineligible patients, even in those with poor PS, not only for achieving sustained remission but also for serving as a palliative measure to optimize the quality of life.

With a long-term follow-up report of the pivotal phase 2 study of BV with a median follow-up period of 71.4 months, the incidence of peripheral neuropathy was 57% (33 of 58 cases) [4]. The median time to onset of grade ≥2 peripheral neuropathy was 2.4 months (range, 0.6-11.3 months). On the other hand, 43% (25 of 58 cases) of patients did not experience peripheral neuropathy during long-term follow-up [4]. In a report evaluating the safety and efficacy of extended administration of BV beyond 16 cycles (24 of median cycles, range 17-42) in patients with relapsed or refractory CD30-positive lymphoma, peripheral neuropathy was the most common adverse event, with 74% of any grade and 11% of grade 3 and more [10]. Grade ≥2 peripheral neuropathy was reported in 11 patients (58%), with a median time to onset of 38 weeks (range, 4.1-106.6) [10]. These findings indicate that BV-induced neuropathy (BVIN) is a relatively early event of treatment, but long-term BV dosing is a major promoting factor. Our patient first developed peripheral neuropathy after 42 cycles and 32 months after BV re-administration. While many cases develop peripheral neuropathy early in treatment, in some cases, the condition does not develop or develops late, as in our case, suggesting some uncovered factors promoting or protecting neuropathy. 

Because microtubules are essential for peripheral nerve function, neuropathy is a predictable side effect of the microtubule-disrupting agent MMAE [11,12]. Dose reduction and interval modification or treatment discontinuation are the only effective measures for BVIN. However, this method may have a negative impact on clinical efficacy. In a clinical trial of 231 patients with moderate-to-severe chemotherapy-induced neuropathy secondary to paclitaxel and oxaliplatin administration, duloxetine, a serotonin-norepinephrine reuptake inhibitor and an important pharmacologic agent recommended by the American Society of Clinical Oncology guidelines, demonstrated clinically meaningful analgesic efficacy [13,14]. In our case, the patient had been receiving long-term duloxetine treatment for DISH before BV monotherapy. The documented efficacy of duloxetine in treating chemotherapy-induced neuropathy and our clinical experience may indicate possible benefits of duloxetine as a BVIN prophylactic agent. The effectiveness of duloxetine in preventing and treating peripheral neuropathy requires further investigation.

PS is widely used to determine the suitability and intensity of chemotherapy. However, because of the heterogeneous health status of older adults and their susceptibility to aging-related vulnerabilities, PS is not sufficiently accurate for defining treatment goals and tailoring treatment intensity. Furthermore, with the advent of new drugs, such as molecularly targeted drugs, the range of treatment options for vulnerable patients is expanding. The ASCO and ESMO guidelines recommend the application of geriatric assessment for elderly patients to avoid the risk of undertreatment or overtreatment [15,16]. The excellent clinical efficacy of BV monotherapy and detailed GA evaluation led to the decision on the reintroduction of treatment, ultimately allowing us to avoid undertreatment.

## Conclusions

Our case highlights that the re-administration and extended dosing of BV monotherapy can be an effective and well-tolerated treatment strategy for patients with relapsed ALCL, including those with poor PS. This therapeutic approach achieved sustained disease control with manageable toxicity over an extended period. These findings indicate that BV monotherapy may be particularly useful for vulnerable patients who demonstrate initial tolerance to the treatment, providing a viable alternative when conventional therapeutic options are limited. Further investigation is necessary to establish the optimal treatment duration and strategies for managing long-term adverse effects in this patient population.
